# A Rapid Cloning Method Employing Orthogonal End Protection

**DOI:** 10.1371/journal.pone.0037617

**Published:** 2012-06-07

**Authors:** Arjen J. Jakobi, Eric G. Huizinga

**Affiliations:** Crystal and Structural Chemistry, Bijvoet Center for Biomolecular Research, Department of Chemistry, Faculty of Science, Utrecht University, Utrecht, The Netherlands; Institut National de la Santéet de la Recherche Médicale, France

## Abstract

We describe a novel *in vitro* cloning strategy that combines standard tools in molecular biology with a basic protecting group concept to create a versatile framework for the rapid and seamless assembly of modular DNA building blocks into functional open reading frames. Analogous to chemical synthesis strategies, our assembly design yields idempotent composite synthons amenable to iterative and recursive split-and-pool reaction cycles. As an example, we illustrate the simplicity, versatility and efficiency of the approach by constructing an open reading frame composed of tandem arrays of a human fibronectin type III (FNIII) domain and the von Willebrand Factor A2 domain (VWFA2), as well as chimeric (FNIII)_n_-VWFA2-(FNIII)_n_ constructs. Although we primarily designed this strategy to accelerate assembly of repetitive constructs for single-molecule force spectroscopy, we anticipate that this approach is equally applicable to the reconstitution and modification of complex modular sequences including structural and functional analysis of multi-domain proteins, synthetic biology or the modular construction of episomal vectors.

## Introduction

The design and construction of DNA sequences by assembly of modular DNA fragments lies at the core of protein engineering. The advent of synthetic biology with associated technological advances in manufacturing of DNA sequences has recently matured *de novo* synthesis of custom genes into an important resource. In spite of these advances, however, there remains a continuous demand for robust and cost-effective alternatives, adaptable by individual laboratories, to aid downstream processing of DNA sequences. This is reflected by the copious and diverse set of elegant tools and strategies that have been developed to facilitate *ad hoc* combinatorial manipulation of DNA sequences and their transfer into episomal vectors by PCR-based [1]–[4], ligation-based [5]–[7], or recombination-based methods [8], [9]. Among the plethora of available techniques, each has its unique advantages and not all individual assembly requirements can be met by a single strategy. For instance, one difficulty encountered by several applications is the recurrent seamless integration of DNA modules into serial or tandem arrays. As one such example, single-molecule force spectroscopy frequently employs repeat proteins to facilitate the identification of characteristic mechanical fingerprints in the experimental force-distance data and to accelerate the generation of statistically relevant datasets. However, only a minor fraction of proteins biologically occur in a repetitive context. Artificial repeat constructs (often referred to as “polyproteins”) are therefore typically obtained by recombinant expression of engineered constructs in which proteins or protein domains are arranged in tandem arrays [10], [11]. At the DNA level, sequential assembly of such constructs by conventional methods typically relies on asymmetric recognition sites for restriction endonucleases [12] or on recycling of restriction sites [2], [13]. These methods either lack control over the exact composition of the assembly product or they require multi-fragment cloning and a series of time-consuming subcloning steps [14]. To accelerate the assembly of such constructs, we recognized the need for a simple and robust strategy permitting the modular recombination of DNA fragments *in vitro*. In multi-step chemical synthesis, efficient strategies have been developed to systematically assemble combinations of molecular building blocks into large assortments of diverse compounds. Surprisingly, several core concepts of these strategies have not yet been adapted to the assembly of recombinant DNA sequences, although the prerequisites for the efficient use of such formats are similar. For instance, chemical synthesis strategies frequently employ transient protecting groups that allow modular building blocks (synthons) to be assembled at specific reactive sites in a defined synthetic sequence. This concept is supported by the ability to selectively remove these protecting groups with a set of mutually exclusive (orthogonal) reaction conditions. In the context of DNA engineering, such molecular building blocks can be defined as DNA synthons and reactive sites as mutually compatible, cohesive overhang sequences that permit DNA ligase-mediated assembly reactions. Likewise, protecting groups may represent peripheral sequences that can be selectively eliminated, by for instance specific restriction enzymes, to reveal cohesive overhang sequences. Directional and recurrent recombination strategies for DNA synthons can accordingly be realized by orthogonal deprotection reactions that expose cohesive overhang sequences at opposite ends of the modules. In the course of any such assembly process, the selective deprotection of defined sequences permits pasting together complementary synthons. To our knowledge there has been no reported gene assembly strategy that exploits the concept of protecting groups for *in vitro* assembly of DNA sequences. We here validate applicability of these principles to the assembly of expression cassettes encoding tandem arrays of protein domains for single-molecule force spectroscopy.

## Results

### General Assembly Strategy

A restriction and ligation-based approach to DNA assembly is introduced that follows the general concept of protecting group-based chemical synthesis strategies. We implement this strategy by designing DNA synthons flanked by protecting groups, which can be selectively removed to expose cohesive overhang sequences. These attributes allow synthons to enter repeated cycles of selective deprotection and fusion reactions *in vitro*, without requiring subcloning steps. As a key concept of our design, any assembly operation performed on an arbitrary pair of synthons will yield an idempotent composite synthon. That is, product synthons may serve as entry synthons for subsequent assembly steps. The resulting versatility of this *split-and-pool* approach allows sequential, parallel and hierarchical strategies for the assembly of DNA modules to be employed with minimal screening efforts.

### Synthon Design

The concept of protecting groups builds on the ability to selectively control the site of modification in a scaffolding synthon, and hence, to precisely define the reaction sequence leading to the final assembly product. For our purpose this requires the introduction of two types of protection groups that can be removed with site-specific (orthogonal) deprotection reactions. Within the framework of DNA assembly this can be readily met by exploiting the excellent specificity of restriction endonucleases towards recognizing and cleaving DNA sequences. In particular, type IIS restriction endonucleases are able to cleave arbitrary sequences at a precise distance from their non-palindromic recognition site and thereby expose user-defined overhang sequences. This unique property of IIS enzymes has the advantage that no artifacts are introduced into the synthons if the recognition site for the enzyme is contained within a flanking sequence that does not become part of the final assembly.

**Figure 1:**
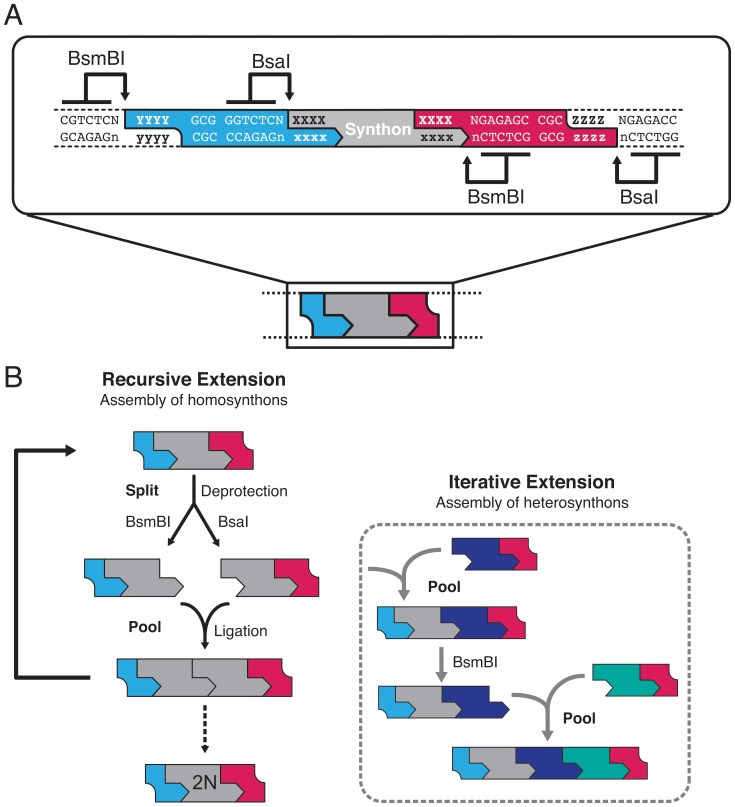
Split-and-pool assembly of DNA synthons. (A) Entry synthons are flanked on both sides by recognition sequences for the type IIS endonucleases BsaI and BsmBI. Restriction by either BsaI or BsmBI selectively exposes user-definable 4-base cohesive overhang sequences (5′-XXXX vs. 5′-xxxx) at one end of the synthon, while maintaining orthogonal protection groups (with 5′-YYYY vs. 5′-zzzz overhangs) at the opposite end. (B) Schematic representation of the ‘split-and-pool’ assembly principle. Cohesive ends of entry synthons are selectively deprotected by digestion with either BsaI or BsmBI. Pooling of the deprotected synthons in the presence of ligase results in unidirectional assembly, affording an idempotent tandem repeat synthon by restoration of orthogonal protecting groups on opposite ends. Each product module can recursively enter the assembly cycle (left panel) N times to yield concatameric synthons with 2N elements. The same strategy can be applied to the assembly of heterosynthons (dashed box), which allows for the engineering of chimeric and multimodular proteins or polycistronic genes.

The application of IIS endonucleases in DNA assembly is not new, and a number of resourceful strategies have recently emerged [5], [7], [15], [16]. We here attempt to extend these methods by introducing a general protecting group strategy that exploits the unique properties of IIS endonucleases. In our general design, the ends of synthons are flanked by oppositely oriented recognition sites for two different IIS endonucleases (**Fig. 1A**). The choice of the endonuclease used for excision of the synthon from an entry vector determines which end of the synthon is protected and which end is reactive/cohesive. The orthogonal motif, together with appropriately selected overhang sequences, supports unidirectional and site-specific assembly of (composite) modules on either end of the entry synthon (**Fig. 1B**). In our design we have chosen IIS endonucleases with 6 base pair recognition sequences, whose theoretical frequency of occurrence is approximately once in 2048 base pairs (4^6^/2), depending on the GC content of the donor genome. This is sufficiently rare to allow application of this approach to typical gene fragments; however, prior mutagenesis may occasionally be required to remove internal sites. From the wealth of commercially available IIS endonucleases, we chose BsmBI and BsaI for reasons of robustness and similarity in buffer and temperature requirements (**Table 1**). The recognition sequences of BsmBI and BsaI differ only in a single nucleotide. Conveniently, another IIS endonuclease, BsmAI, recognizes the common subset of these recognition sequences, so that one can optionally combine both cleavage reactions to yield synthons that are deprotected on both ends.

**Table 1:** Buffer and temperature preferences of the BsmBI/BsaI/BsmAI system.

Endonuclease	RecognitionSequence	NEB buffer	T (°C)
**BsaI**	**GGTCTC** *N/NNNN*	4 or 3	50
**BsmBI**	**CGTCTC** *N/NNNN*	3	55
**BsmAI**	**GTCTC** *N/NNNN*	3	50

The IIS endonucleases selected generate 4-base 5′-overhangs. The optimal sequences for these overhangs will depend on the purpose of the particular assembly design, but several factors should be carefully considered. First, palindromic overhang sequences carry the risk of unintended self-ligation. Second, GC-rich sequences are generally favored in compatible overhangs for their increased annealing efficiency. This is particularly important in the context of the short reaction times used in our *in vitro* assembly strategy. Third, for designs related to the manipulation of sequences such as tandem domain assemblies, the choice of sequences for the junctions primarily depends on which amino acid sequences will most likely be tolerated within the domain linker. While we purposely introduced scar sequences in the application of the strategy reported here, the design in principle supports seamless assembly formats if the cohesive sequences are chosen appropriately.

Protecting groups also carry 4-base overhang sequences because they are excised together with the synthon from an entry vector. These overhangs must comply with the requirement of being non-palindromic, mutually non-complimentary and, at the same time, orthogonal to the cohesive assembly overhangs.

### A Split-and-Pool Strategy for Rapid Construct Assembly

**Figure 2:**
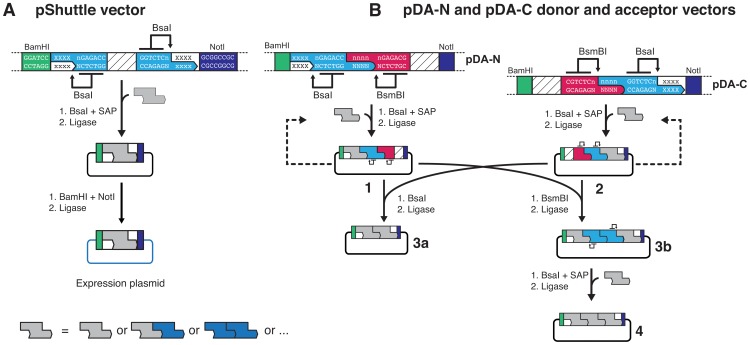
Vectors for synthon recombination and transfer to expression plasmids. (A) Deprotected synthons can be ligated into a BsaI-digested shuttle vector (pShuttle) that contains 5′-BamHI and 3′-NotI restriction sites compatible with our in-house collection of expression vectors. (B) For applications that require modular recombination or insertion of individual elements, synthons can be ligated into the modular assembly vectors pDA-N or pDA-C. pDA-N vectors that carry synthons encoding amino-terminal elements of protein constructs (**1**) can be combined with synthons encoding carboxyl-terminal elements from pDA-C vectors (**2**) to yield a composite product (**3a**), optionally leaving an additional entry point via BsaI restriction sites to insert additional synthons (**3b**). Final assemblies (**3a** and **4**) contain 5′-BamHI and 3′-NotI restriction sites for transfer into expression vectors.

The assembly procedure consists of three stages. First, entry vectors are digested with either of the two IIS endonucleases such that in each case the resulting entry synthon has a 4-base cohesive overhang exposed at one end while one of the orthogonal protecting groups is retained at the opposite end (**Fig. 1B**). Subsequently, complementary entry synthons are pooled and fused by ligation, yielding composite synthons that are idempotent to the entry synthons by restoration of the orthogonal protecting group configuration. At each assembly level, the synthons are digested in parallel with one of the IIS endonucleases essentially as in the first step. The procedure is reminiscent of *split-and-pool* assembly strategies frequently applied in combinatorial chemistry. Within this framework synthons can enter iterative or recursive assembly cycles until the required target construct is obtained (**Fig. 1B**). Since the assembly of orthogonally deprotected synthons is unidirectional, only a single product can be formed. Finally, the assembled product synthons can be ligated back into the empty entry vector, which may serve as a general repository vector (see **Fig. 1A**). Alternatively, the synthons can be directly cloned into a shuttle vector (pShuttle) for subsequent transfer into expression plasmids (**Fig. 2A**). In our laboratory we utilize a standardized set of expression vectors that uniformly contain 5′-BamHI and 3′-NotI restriction sites to facilitate rapid subcloning of constructs into vectors for different expression hosts and are equipped with a variety of tag- or signal peptide decorations. The pShuttle vector is a modified pCR8-TOPO vector carrying a stuffer sequence that is flanked on both sites by BsaI restriction sites, which create cohesive overhangs compatible with those from the deprotected synthons; and additional 5′-BamHI and 3′-NotI restriction sites compatible to our in-house library of expression vectors.

### Donor and Acceptor Vectors for Facile Synthon Shuffling

Common to most concatamerization strategies, one technical limitation arising from our assembly design is that it does not support the exchange or insertion of synthons in the final assembly product. We realized that such additional versatility can be added by introducing modular assembly vectors and therefore designed a compatible pair of donor and acceptor vectors (pDA-N and pDA-C) that support modular recombination of synthons and insertion of synthons into a nearly completed assembly product (**Fig. 2B**). Similar to the pShuttle vector, these vectors carry a stuffer sequence flanked by restriction sites for IIS endonucleases to create overhang sequences compatible with the overhangs of deprotected synthons. Synthons can be subcloned into pDA-N and pDA-C vectors, which can either serve as the source of donor synthons or act as acceptor vectors but differ in the relative position, amino- or carboxyl-terminal, of the synthons that they contribute to the translated assembly product (**Fig. 2B** **,** product 1 and 2). Our design supports two alternative strategies: (i) Recombination of donor and acceptor vectors using the BsaI restriction site together with the BamHI or NotI restriction sites as appropriate, leads to products that have defined 5′- and 3′-synthons (**Fig. 2B** **,** product 3a). (ii) Recombination of donor and acceptor vectors using the BsmBI restriction site together with the BamHI or NotI restriction sites as appropriate, leads to products that have defined 5′- and 3′-synthons, while retaining two BsaI sites that provide an additional entry point (**Fig. 2B** **,** product 3b). As an example, this design supports the rapid insertion of proteins of interest to be sandwiched between fingerprint domains as used frequently in single-molecule force spectroscopy, or the one-step modification of open reading frames by insertion of mutant domains (**Fig. 2B** **,** product 4).

### Assembly of ^13^FNIII and von Willebrand Factor A2 Domain Tandem Repeats

**Figure 3:**
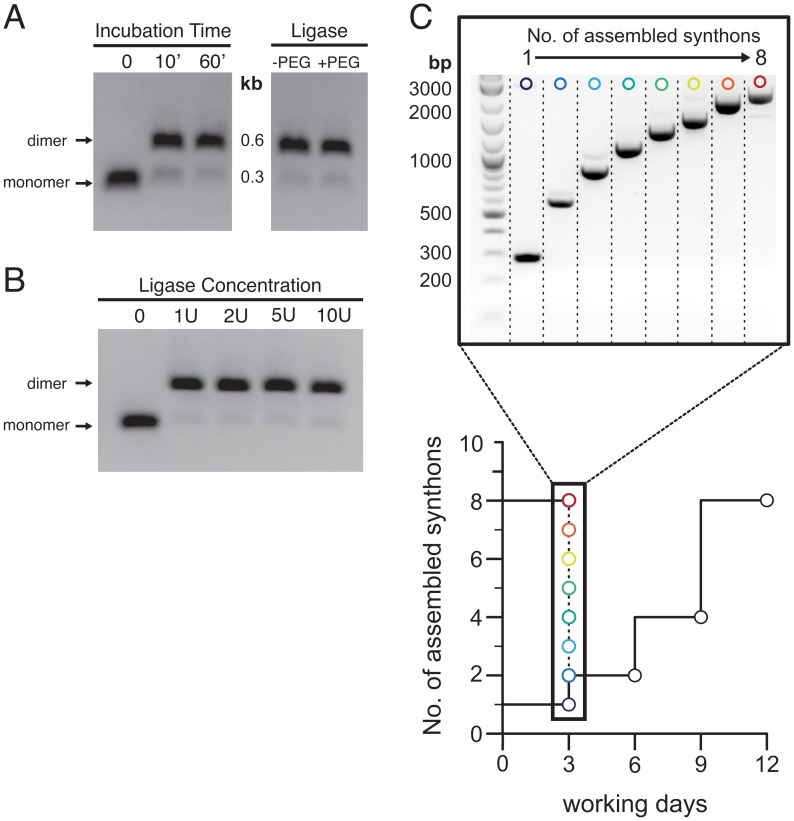
Efficient synthon assembly with split-and-pool reactions. (A) Equimolar amounts of BsaI or BsmBI deprotected ^13^FNIII synthons were incubated with 1 unit of T4 ligase and product formation was assessed at different time points (left panel) or after 15 min in buffer conditions with and without 15% (w/v) PEG6000 (right panel). (B) No significant differences in assembly efficiency are observed after 15′ incubation at ligase concentrations ranging from 1 to 10 units. (C) Performance of split-and-pool assembly in comparison to sequential approaches. Within one day the comprehensive series of (^13^FNIII)_1_ to (^13^FNIII)_8_ repeats can be assembled with the split-and-pool approach (spectrum circles) and ligated into the pShuttle vector. After a single cloning step expression plasmid is obtained on day 3. In comparison, sequential assembly with e.g. the BamHI/BglII system requires 12 days to obtain the (^13^FNIII)_8_ construct.

We tested the applicability of our approach during the construction of a series of expression cassettes harboring tandem arrays of homologous protein domains for single-molecule force spectroscopy. DNA synthons encoding Asn1813 to Thr1901 of a fibronectin type III (^13^FNIII) domain of human fibronectin [17] were prepared according to the design principles outlined in **Fig. 1A**. The flanking sequences were chosen such that digestion with BsaI produces a 5′-GGGG overhang capable of annealing with a 5′-CCCC overhang present on synthons restricted with BsmBI. The ligation site between synthons translates to a GlyGly sequence in the final protein. BsaI and BsmBI restricted synthons retain a protection group on one end with orthogonal 5′-AAAA overhangs. Entry synthons were processed during three recursive assembly cycles. Relatively short reaction times (10–15 min) at ligase concentrations of 1 unit sufficed to covalently link complementary synthons with excellent yield (**Fig. 3A,B** **).** We note, however, that we did not assess the efficiency of our approach with overhang sequences other than the cohesive GGGG/CCCC system exemplified in this report.

Using this protocol it proved feasible, in one working day, to assemble the series of constructs comprising one up to eight ^13^FNIII synthons (denoted (^13^FNIII)_1_ through (^13^FNIII)_8_; **Fig. 3C**). The cumulative yield loss resulting from repeated restriction-ligation cycles and associated gel purifications prohibited the assembly of even larger constructs. This should be possible, however, by increasing the amount of starting material. Alternatively, selected assemblies could be ligated back into the empty entry vector and amplified in bacterial cultures for further processing (see **Fig. 1**). Compared to conventional methods based on sequential subcloning (e.g. BamHI/BgIII system), our approach saved at least 9 days to yield the largest (^13^FNIII)_8_ construct (**Fig. 3C**). Finally, the composite synthons were ligated into the pShuttle vector, as well as the donor/acceptor vectors pDA-N and pDA-C from which they may be recombined with other synthons to yield heterocomposite constructs (see below).

In a similar fashion we constructed multimeric constructs containing tandem repeats encoding residues Val1478 to Gly1674 of the human von Willebrand factor A2 domain (VWFA2). In this case we used the donor and acceptor vector system to clone the assembled (A2)_2_, (A2)_4_ and (A2)_6_ repeats into both of the pDA-N and pDA-C vectors and then combined the appropriate donor synthons and acceptor vectors (see **Fig. 2B**) to construct the final (A2)_6_, (A2)_8_ and (A2)_10_ assemblies for recombinant protein expression. The VWFA2 domain has a roughly spherical shape of ∼30 Å diameter with the amino- and carboxyl-termini protruding from the same side of the domain [18], [19]. To facilitate independent folding and to prevent steric clashes between adjacent domains during stretching in single-molecule experiments, we engineered (GlySer)_3_ repeats on either side of the domain. The resulting 12-residue linker, together with amino- and carboxyl-terminal residues that appear unstructured form the crystal structure [18], leads to an average end-to-end distance of at least 16 Å between the termini of adjacent domains in the concatameric construct as estimated by the worm-like chain model [20].

**Figure 4:**
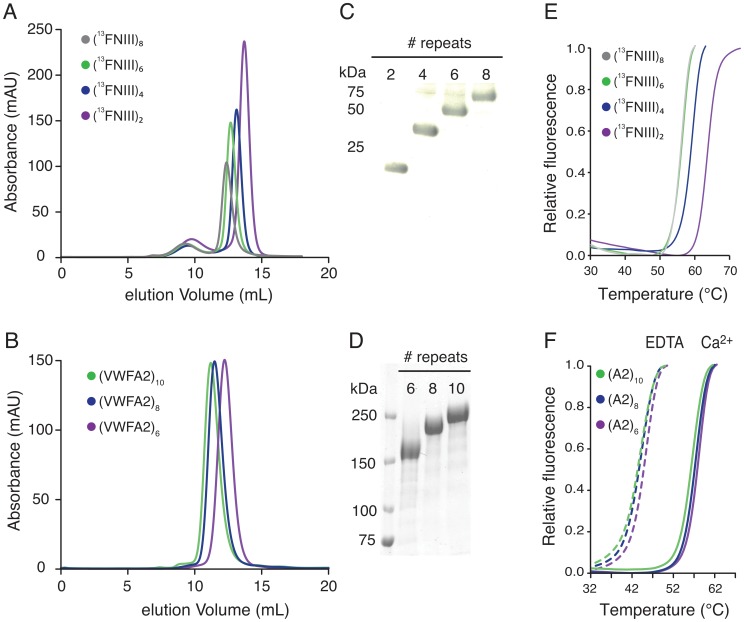
Expression and characterization of ^13^FNIII and VWFA2 tandem repeat proteins. Superposed elution profiles from size exclusion chromatography of (^13^FNIII)_2–8_ proteins (A) and (VWFA2)_6–10_ (B). (C,D) Coomassie stained SDS-PAGE of the purified proteins. (E,F) Unfolding curves from Thermofluor analysis suggest that the concatameric constructs are properly folded. Note the consistent shift of the (VWFA2)_n_ unfolding curves in the presence and absence of Ca^2+^.

To verify our assembly format, constructs (^13^FNIII)_2,4,6,8_ and (A2)_6,8,10_ were expressed in HEK293-EBNA1 (HEK-E) cells and purified by immobilized metal affinity chromatography followed by size exclusion chromatography (**Fig. 4A-D**). We performed thermal denaturation assays to assess the folding state of the purified proteins and to investigate the effect of repeat number on thermal stability. All constructs display a two-state unfolding transition from a stable fluorescence baseline (**Fig. 4E,F**). We observed a gradual decrease in melting temperature (T_m_) with increasing number of tandem repeats for the concatameric (^13^FNIII)_n_ constructs (Table 2). Consistently, the unfolding temperature of 62.3±0.2°C for the dimeric ^13^FNIII construct is lower than the value of 72°C reported for the monomer [21]. In contrast, the series of concatameric VWFA2 constructs showed little variation in thermal stability. We have previously reported on the modulation of thermodynamic and mechanical stability of VWFA2 by binding of Ca^2+^ to a highly conserved calcium-binding site [18]. In accordance, we find a marked T_m_ shift for buffer conditions containing either Ca^2+^ or EDTA for the tandem domain constructs, indicating that the VWFA2 domains fold into their native structure also in this repetitive context.

**Table 2:** Apparent melting temperature of concatameric proteins.

Construct	T_m_ (°C)
(^13^FNIII)_2_	62.3±0.2
(^13^FNIII)_4_	57.4±0.3
(^13^FNIII)_6_	54.5±0.1
(^13^FNIII)_8_	54.2±0.1
(VWFA2)_6_ + Ca^2+^	57.7±0.1
(VWFA2)_8_ + Ca^2+^	57.0±0.1
(VWFA2)_10_ + Ca^2+^	56.1±0.1
(VWFA2)_6_ + EDTA	44.7±0.1
(VWFA2)_8_ + EDTA	43.3±0.3
(VWFA2)_10_ + EDTA	43.1±0.1

Recombinant expression of repetitive DNA sequences carries the risk of genetic instability, potentially resulting in synthesis of truncated or rearranged protein chains. Contrary to our concerns, we found very little contaminants in the supernatant of expression cultures with a molecular weight different from that expected from the transfected DNA construct (**Fig. 4C,D** **and Fig. S1**). Although we did not perform transcriptional analysis to assess whether these contaminating bands are a consequence of genetic recombination or proteolysis, our data indicates genetic instability is not a major problem in HEK-E cells for our constructs.

### Assembly of Chimeric (^13^FNIII)_2_-VWFA2-(^13^FNIII)_2_ Constructs

**Figure 5:**
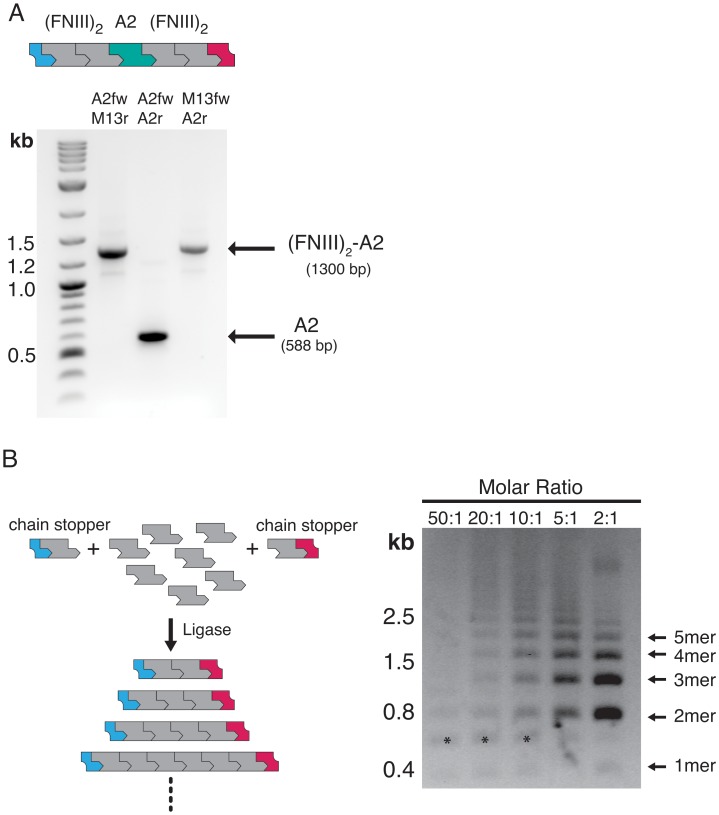
Assembly of chimeric constructs and one-pot concatamer formation. (A) Assembly of (FNIII)_2_-VWFA2-(FNIII)_2_ sandwich constructs from a modular assembly vector (top). PCR amplification with specific primers (indicated above the lanes; **Table S1**) show that the A2 synthon is sandwiched between two ^13^FNIII repeats. (B) One-pot concatamer formation with orthogonal chain stoppers. Fully deprotected synthons are mixed in different molar ratios with orthogonal chain stoppers (equivalent synthons with protecting groups on one end). Increasing the concentration of chain stoppers shifts the size distribution towards shorter concatamers. Molar ratios of unprotected synthons:chain stoppers are indicated at the top of the lanes. Bands marked with asterisks presumably correspond to circularized dimers.

Repetitive constructs are frequently used in single-molecule experiments for reasons of accelerated data sampling and confidence in distinguishing specific tethers from non-specific background. However, such constructs can hamper the identification of intermediates owing to simultaneous unfolding of structural elements in other domains of the repetitive context. In such cases, the unambiguous assignment of unfolding traces is facilitated if individual domains of interest are sandwiched between a repetitive series of protein domains whose elastic properties are well characterized, such as immunoglobulin-like domains of titin [13], [14], *Dyctostelium discoideum* filamin (ddFLN) [22] or fibronectin FNIII domains [17]. Our donor and acceptor vectors support the rapid assembly of such constructs and we illustrate this process by sandwiching single VWFA2 domains between amino- and carboxyl-terminal ^13^(FNIII)_2_ repeats (**Fig. 5A**). For this purpose, a ^13^(FNIII)_2_-X-^13^(FNIII)_2_ vector construct was created that contains inversely oriented BsaI sites between the ^13^FNIII repeats to form the entry point for synthon insertion (**see** **Fig. 2B**). ^13^(FNIII)_2_ synthons were subcloned into both pDA-N and pDA-C vectors and subsequently recombined by ligating the BsmBI/BamHI fragment from the resulting pDA-N-^13^(FNIII)_2_ vector (acting as the donor) into the BsmBI/BamHI-linearized pDA-C-^13^(FNIII)_2_ vector (acting as the acceptor). A synthon encoding residues Val1478 to Gly1674 of the VWFA2 domain and flanking (GlySer)_3_ linkers was subsequently inserted between the ^13^(FNIII)_2_ repeats using the BsaI restriction sites. The successful assembly was verified by PCR (**Fig. 5A**).

## Discussion

We have demonstrated that the application of IIS restriction endonucleases can be readily combined with a simple protecting group concept to create a versatile framework for the rapid assembly of modular DNA building blocks into functional gene constructs. Several features distinguish our approach from previously reported strategies: Contrary to conventional restriction enzyme or recombination-based methods that require subcloning steps between consecutive assembly cycles, the application of enzymatically cleavable protecting groups and the restoration of idempotent composite synthons permits assembly of any arbitrary sequence of modular DNA units into a functional gene product *in vitro*. As we illustrate with the construction of ^13^FNIII and VWFA2 tandem repeats, this approach considerably accelerates the assembly procedure. One detriment that comes with our strategy is that it requires repeated cycles of gel purification, making it labor-intensive and, as of yet, unsuitable for automation. Considering the remarkable efficiency of the ligation reactions that we observed in the assembly steps process, one could consider omitting the purification steps and submit samples to direct split-and-pool reaction cycles. This will be applicable only if the project concerns the construction of a series of homologous repeats, since obviously even spurious reactive contaminants are unacceptable if a defined set of heterogeneous synthons is to be assembled into a particular sequence.

Several multi-fragment strategies [5], [7], [16], [23] that have been recently developed to address a similar scenario depend on a distinct pair of cohesive overhang sequences for each sub-fragment. Due to restricted redundancy of the genetic code, this becomes a limitation if the amino acid sequences of the linkers between domains are to be identical. The concept of orthogonal protecting groups straightforwardly bypasses these constraints and linker sequences can be readily standardized by appropriate primer design. Common to all cloning strategies that rely on restriction enzymes, however, our approach is hampered by the probability of the recognition sequences to occur within the target gene fragments. Although these sites may be removed by silent mutagenesis or excluded *a priori* by employing custom-designed synthetic gene units, this may represent a limitation regarding applications that involve larger DNA modules. Alternative to the presented sequential assembly strategy, mixtures of products with a varying repeat number can be obtained by mixing fully deprotected synthons with singly deprotected synthons acting as orthogonal chain stopper units. Depending on the stochiometric ratios used for these reactions, libraries of composite modules can be constructed in a one-pot reaction (Fig. 5B).

We introduce further versatility by establishing a three-point entry vector system based on donor and acceptor vectors. These vectors form a cassette framework for the rapid modification of open reading frames to revise assemblies that have failed in recombinant expression trials, the insertion of mutated or truncated synthons or to sandwich target domains between internal standards in force spectroscopy applications.

### Perspective

The directional *in vitro* assembly strategy based on orthogonal protecting groups is robust, technically simple and can be readily tailored to suit individual assembly scenarios. We anticipate that our method will significantly facilitate and accelerate protein engineering for single-molecule force spectroscopy experiments, and may find broader applicability in other settings involving the reconstitution and modification of complex modular sequences.

### Supporting Information

This text contains supplementary figures.

## Methods

### General

Sequences of oligonucleotides are provided in Table S1. All entry synthons and vectors were verified by DNA sequencing.

### Preparation of DNA Synthons

A DNA fragment encoding residues 1813-1901 of human ^13^FNIII (UniProt accession P02751) flanked by 5′-BsmBI-BsaI and 3′-BsaI-BsmBI restriction sites was optimized for mammalian codon usage, RNA structure, and GC content and synthesized (DNA 2.0, Menlo Park, CA). Overhang sequences were chosen as AAAA for the protection group overhangs and GGGG for the cohesive overhangs in the 5′-BsmBI-BsaI sequence vs. AAAA and CCCC for protection group and cohesive overhangs in the 3′-BsaI-BsmBI sequence (compare **Fig. 1A**). To preserve the reading frame the 3′ CCCC bases are preceded by two additional C nucleotides. A DNA fragment encoding residues 1478-1674 of the human VWFA2 domain (UniProt accession P04275; p.V1565A polymorphism) and containing the same overhang sequences was constructed as described [18] using oligos VWFA2.fw and VWFA2.rev. A silent mutation was introduced by QuikChange mutagenesis to remove the internal BsmBI site in the A2 fragment. Both constructs were cloned into pCR8-TOPO (Invitrogen) for DNA amplification.

### Construction of Shuttle and Donor/acceptor Vectors

A DNA stuffer fragment encoding part of the VWF cDNA was amplified by PCR with oligos containing a 5′-BamHI-BsaI-BsmBI sequence for pDA-N (oligos pDA-N.fw and pDA-N.rev) or a 5′-NotI-BsaI-BsmBI sequence for pDA-C (oligos pDA-C.fw and pDA-C.rev), respectively. The PCR products were then cloned into pCR8-TOPO and a fragment of the stuffer sequence containing internal BsaI sites was excised by NsiI digestion to create the donor/acceptor vectors pDA-N and pDA-C, respectively. The plasmid backbone of pCR8-TOPO does not contain internal BsaI, BsmBI or BsmAI sites. Similarly, the pShuttle vector was obtained from PCR amplification of the stuffer fragment using oligos pShuttle.fw and pShuttle.rev.

### Assembly of ^13^FNIII and VWFA2 Repeats

Plasmid DNA of pCR8-^13^FNIII and pCR8-VWFA2 was prepared from 50 mL o/n cultures using a Midiprep kit (Sigma Aldrich) and concentrated by ethanol precipitation to obtain ∼0.7 mg DNA at 1.5–2.5 µg/µl. 50–100 µg DNA were mixed with 10x NEB reaction buffer 3 and either 5 µl BsaI or BsmBI (New England Biolabs) in a total volume of 50–100 µl and incubated at 50°C for 1 h on a thermal cycler (Bio-Rad S1000). After a 30 min gel electrophoresis run on 2% (w/v) agarose gels, synthon fragments were excised and gel purified using a DNA gel purification kit (Promega). Usually around 10.5 µg of (^13^FNIII)_mono_ synthon were recovered (∼ 85 % yield). Equal molar amounts (typically 250–500 ng at ∼ 100 – 250 ng/µl ) of orthogonally protected synthons were mixed, 0.5–1 unit T4 ligase (Fermentas) and T4 ligase buffer (Fermentas) were added and the ligation mixture was incubated for 10–20 min at 16°C. Adding additional ligase had little effect on ligation efficiency. A 30 min gel electrophoresis run on 0.8–1.5 % (w/v) agarose gels provided sufficient separation of product from unreacted synthons. Product synthons were gel purified as described above and split into two equal volumes, which were treated with either BsaI or BsmBI to yield orthogonally protected dimers. This assembly process was repeated until the target constructs were obtained. For further processing, the target synthons were digested for 30 min with BsaI and BsmBI to deprotect both ends and purified over a DNA purification spin column (Promega). 1 µg pShuttle, pDA-N and pDA-C vectors were digested with BsaI for 1 h at 50°C after which the vectors were dephosphorylated by the addition of 0.5 units shrimp alkaline phosphatase (SAP, Fermentas) for 1 h at 37°C. We found that self-ligation could be significantly reduced by repeating this treatment, without adding new BsaI endonuclease. (FNIII)_n_ synthons were ligated directly into the BsaI-linearized pShuttle vector whereas (A2)_n_ constructs were ligated into pDA-N and pDA-C donor and acceptor vectors using low vector concentrations of 0.3–0.6 ng/µl and vector:insert ratios below 4∶1, which we found to reduce synthon self-assembly.

### Assembly of (FNIII)_2_-(VWFA2)-(FNIII)_2_ Constructs

(FNIII)_2_ synthons were assembled as described above and ligated into BsaI-linearized pDA-N and pDA-C vectors. Next, the (FNIII)_2_ synthon was digested from the pDA-N-(FNIII)_2_ vector (3 µg) using BsmBI and BamHI and ligated into the BsmBI/BamHI-linearized pDA-C-(FNIII)_2_ acceptor vector. The resulting vector pDA-(FNIII)_2_-X-(FNIII)_2_ was linearized with BsaI to create an entry point for the A2 synthon. The BsmBI/BsaI deprotected A2 synthon was subsequently ligated into the linearized vector to create the chimeric pDA-(FNIII)_2_-A2-(FNIII)_2_ construct.

### Protein Expression and Purification

Tandem repeat constructs of ^13^FNIII and VWF-A2 were ligated into the BamHI and NotI sites of a modified pTT3 vector carrying a cystatin S signal peptide, an amino-terminal hexahistidine tag and a carboxyl-terminal biotin acceptor peptide sequence (GLNDIFEAQKIEWHE). Human embryonic kidney cells stably expressing EBNA1 were transiently transfected as described [24]. Proteins were purified by Ni-sepharose affinity chromatography (GE Healthcare), followed by size exclusion chromatography in 20 mM HEPES (pH 7.5), 150 mM NaCl on Superdex 200 10/300 column (GE Healthcare). Proteins were concentrated to 1 mg/mL and stored at −80°C.

### Thermal Denaturation Assays

Thermal denaturation assays were performed essentially as described [18]. Briefly, 6.25 µl of 10x Sypro Orange (Sigma-Aldrich) was mixed with 6.25 µl of a 0.4 mg/mL protein solution in 20 mM HEPES (pH 7.5), 150 mM NaCl and immediately mixed with 12.5 µl of assay buffer containing 50 mM HEPES (pH 7.5), 150 mM NaCl for ^13^FNIII concatamers or assay buffer containing additionally 1 mM CaCl_2_ or 5 mM EDTA for VWFA2 concatamers. The apparent T_m_ of individual unfolding curves (n = 3) was determined from a sigmoidal fit to the normalized fluorescence data [25].

## Supporting Information

Figure S1 **Western blot of (VWFA2)_n_ in expression medium demonstrating minor contamination by concatamers with a molecular weight different from the target construct (indicated by arrows).** These contaminants may either result from genetic recombination or proteolysis. Horse radish peroxidase-coupled α-VWF (Dako) was used to stain the blot.
(TIF)

Table S1 **Oligonucleotide sequences.**
(DOCX)
