# Supplementary figures and images for: A Rapid Cloning Method Employing Orthogonal End Protection
Source: PLoS One. 2012 Jun 7;7(6):e37617. doi: 10.1371/journal.pone.0037617 (PMC3369885; doi:10.1371/journal.pone.0037617)

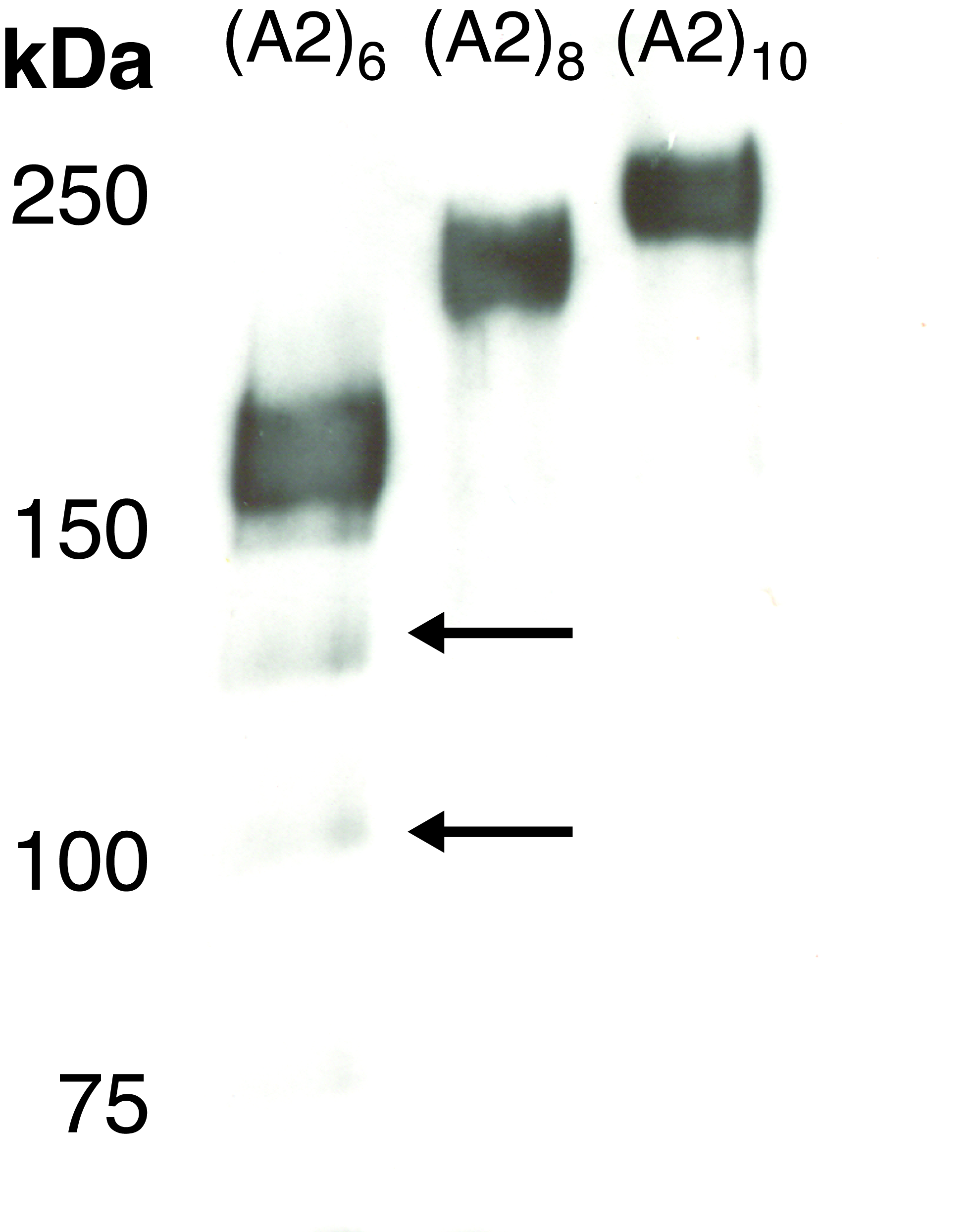

Supplement: Figure S1 — Western blot of (VWFA2)n in expression medium demonstrating minor contamination by concatamers with a molecular weight different from the target construct (indicated by arrows). These contaminants may either result from genetic recombination or proteolysis. Horse radish peroxidase-coupled α-VWF (Dako) was used to stain the blot. (TIF) [file pone.0037617.s001.tif]
