# Supplementary material for: A Rapid Cloning Method Employing Orthogonal End Protection
Source: PLoS One. 2012 Jun 7;7(6):e37617. doi: 10.1371/journal.pone.0037617 (PMC3369885; doi:10.1371/journal.pone.0037617)
Supplement: Table S1 — Oligonucleotide sequences. (DOCX) [file pone.0037617.s002.docx]

**Table S1** Oligonucleotide sequences

| **oligo** | **Sequence (5’ 🡪 3’)** |
| --- | --- |
| VWF-A2.fw | CGTCTCAAAAAGCGGGTCTCGGGGGAGTGGTAGTGGTTCTgtgggcccggggctcttggg |
| VWF-A2.rev | GGTCTCAAAAAGCGCGTCTCCCCCCCCACTTCCACTACCcccctctccggagcagcacc |
|  |  |
| pShuttle.fw | GGATCCGGCAGCGGGGGGGGAGACCCCTCCTTCACTCGGACACACACC |
| pShuttle.rev | GCGGCCGCACTACCCCCCCGAGACCGCTAGCTATTTTGATGAAATGAAGAGTTTCGCC |
|  |  |
| pDA-N.fw | GGATCCGGCAGCGGGGGGGGAGACCCGCCACACGAGACGCCTCCTTCACTCGGACACACACC |
| pDA-N.rev | CACTGCATTCTAGTTGTGGTTTGTCCAAACTC |
|  |  |
| pDA-C.fw | GTTGCCTGACAACGGGCCACAAC |
| pDA-C.rev | GCGGCCGCACTACCCCCCCGAGACCGCGTGTGTGAGACGGCTAGCTATTTTGATGAAATGAAGAGTTTCGCC |
